# Supplementary material for: Genome-Wide and Functional View of Proteolytic and Lipolytic Bacteria for Efficient Biogas Production through Enhanced Sewage Sludge Hydrolysis
Source: Molecules. 2019 Jul 18;24(14):2624. doi: 10.3390/molecules24142624 (PMC6680700; doi:10.3390/molecules24142624)
Supplement: Supplementary file 1 [file molecules-24-02624-s001.pdf]

Supplementary Material

Genome-wide and functional view of proteolytic and lipolytic bacteria for efficient biogas production through enhanced sewage sludge hydrolysis

Krzysztof Poszytek [1], Joanna Karczewska-Golec [1], Mikolaj Dziurzynski [2], Olga Stepkowska-Kowalska [2], Adrian Gorecki [2], Przemyslaw Decewicz [2] , Lukasz Dziewit [2] and
Lukasz Drewniak [1]*

[1] Laboratory of Environmental Pollution Analysis, Faculty of Biology, University of Warsaw, Miecznikowa 1, 02-096 Warsaw, Poland; kposzytek@biol.uw.edu.pl, karczewska@biol.uw.edu.pl, ldrewniak@biol.uw.edu.pl

[2] Department of Bacterial Genetics, Institute of Microbiology, Faculty of Biology, University of Warsaw, Warsaw, Poland; mikolaj.dziurzynski@biol.uw.edu.pl, olga.stepkowska@gmail.com, agorecki@biol.uw.edu.pl, decewicz@biol.uw.edu.pl, ldziewit@biol.uw.edu.pl

* Correspondence: ldrewniak@biol.uw.edu.pl; Tel.: +48-225-541-219

Strain	Molasses									Dairy Waste									Malt Extract								
	0.1%			0.5%			1.0%			0.1%			0.5%			1.0%			0.1%			0.5%			1.0%		
	0h	24h	48h	0h	24h	48h	0h	24h	48h	0h	24h	48h	0h	24h	48h	0h	24	48	0h	24h	48h	0h	24h	48h	0h	24h	48h
Rummeliibacillus sp. POC 4	0.05	0.10	0.11	0.07	0.14	0.17	0.10	0.18	0.34	0.04	0.10	0.08	0.05	0.27	0.20	0.07	0.38	0.50	0.05	0.05	0.06	0.05	0.06	0.07	0.07	0.07	0.09
Ochrobactrum sp. POC 9	0.06	0.11	0.22	0.05	0.17	0.19	0.06	0.25	0.29	0.05	0.05	0.07	0.06	0.09	0.09	0.07	0.35	0.52	0.05	0.07	0.08	0.05	0.10	0.10	0.06	0.13	0.15
Brevundimonas sp. LPMIX 5	0.05	0.08	0.10	0.05	0.12	0.14	0.06	0.21	0.31	0.05	0.05	0.06	0.05	0.23	0.31	0.07	0.53	0.48	0.04	0.05	0.05	0.05	0.06	0.06	0.06	0.09	0.09

OD_{600} nm	0.01-0.10	0.11-0.20	0.21-0.30	0.31-0.40	0.41-0.50	0.51-0.60

Figure S1. Bacterial growth on waste substrates**.** Results of three biological replicate are presented, standard deviation were less than 10% and are omitted for clarity.

Table 1. Genome assembly table.

Strain	N50 [bp]	Essential genes present	Missing essential genes	rRNA copy number	Number of predicted genes	GC content [%]	Number of Scaffolds
Rummeliibacillus sp. POC4	*58116*	106/111	TIGR00388: glyQ: glycine--tRNA ligase, alpha subunit.<br>TIGR00408: proS_fam_I: proline--tRNA ligase.<br>TIGR00471: pheT_arch: phenylalanine--tRNA ligase, beta subunit.<br>TIGR00775: NhaD: Na+/H+ antiporter, NhaD family.<br>TIGR02387: rpoC1_cyan: DNA-directed RNA polymerase, gamma subunit.	*1*	*3652*	*34,47*	*164*
Ochrobactrum sp. POC9	*182332*	105/111	TIGR00389: glyS_dimeric: glycine--tRNA ligase.<br>TIGR00408: proS_fam_I: proline--tRNA ligase.<br>TIGR00471: pheT_arch: phenylalanine--tRNA ligase, beta subunit.<br>TIGR00775: NhaD: Na+/H+ antiporter, NhaD family.<br>TIGR01030: rpmH_bact: ribosomal protein L34<br>TIGR02387: rpoC1_cyan: DNA-directed RNA polymerase, gamma subunit.	*1*	*4728*	*55,97*	*255*
Brevundimonas sp. LPMIX5	*224500*	105/111	TIGR00389: glyS_dimeric: glycine--tRNA ligase.<br>TIGR00408: proS_fam_I: proline--tRNA ligase.<br>TIGR00471: pheT_arch: phenylalanine--tRNA ligase, beta subunit.<br>TIGR00775: NhaD: Na+/H+ antiporter, NhaD family.<br>TIGR01030: rpmH_bact: ribosomal protein L34.<br>TIGR02387: rpoC1_cyan: DNA-directed RNA polymerase, gamma subunit.	*1*	*2939*	*66,88*	*97*

Table S2. Average aminoacids identity (AAI) values computed using MIGA webserver.

Strain	Reference strain	AAI [%]	Fraction of genome shared [%]
Rummeliibacillus sp. POC4	*Rummeliibacillus stabekisii* NZ CP014806	*72,81*	<70
	Kurthia sp. 11kri321 NZ CP013217	*65,96*	<70
	Kurthia zopfii NZ LR134474	*64,62*	<70
	Lysinibacillus fusiformis NZ CP010820	*61,54*	<70
	Lysinibacillus sp. 2017 NZ CP029002	*59,97*	<70
Ochrobactrum sp. POC9	*Ochrobactrum anthropi* NZ CP008820	*94,35*	81,03
	Ochrobactrum anthropi ATCC 49188 NC 009667	*93,97*	79,8
	Brucella melitensis NZ CP018492	*84,98*	82,55
	Ochrobactrum pseudogrignonense NZ CP015775	*81,83*	70,11
	Ochrobactrum sp. A44 NZ CP022604	*81,54*	72,14
Brevundimonas sp. LPMIX5	*Brevundimonas naejangsanensis* NZ CP015614	*93,67*	84,46
	Brevundimonas diminuta NZ CP021995	*89,78*	84,93
	Brevundimonas naejangsanensis NZ CP032707	*86,7*	80,23
	Brevundimonas sp. NZ CP015511	*66,51*	<70
	Brevundimonas sp. LM2 NZ CP019508	*62,73*	<70

Table S3. Average nucleotide identity (ANI) values. With minimal threshold set at 80% identity, there were no ANI hits for Rummeliibacillus sp. POC4 strain.

Strain	Reference strain	ANI [%]	Fraction of genome shared [%]
Ochrobactrum sp. POC9	*Ochrobactrum anthropi* NZ CP008820	*90,89*	80,07
	Ochrobactrum anthropi ATCC 49188 NC 009667	*90,87*	74,48
	Brucella melitensis NZ CP018492	*82,74*	69,17
	Ochrobactrum pseudogrignonense NZ CP015775	*80,91*	47,5
	Ochrobactrum sp. A44 NZ CP022604	*80,5*	41,38
Brevundimonas sp. LPMIX5	*Brevundimonas naejangsanensis* NZ CP015614	*93,4*	82,63
	Brevundimonas diminuta NZ CP021995	*90,59*	73,94
	Brevundimonas naejangsanensis NZ CP032707	*89,22*	75,9

Table S4. Heavy metal resistance genes identified within genomes of the Brevundimonas sp. LPMIX5, Rummeliibacillus sp. POC4 and Ochrobactrum sp. POC9 strains.

Gene Name	Predicted Protein Function	Localization (Genbank acc. Numbers)		
		Brevundimonas sp. LPMIX5	Rummeliibacillus sp. POC4	Ochrobactrum sp. POC9
chrA	Export of Cr(VI)	-	1) QWUA01000024.1 - locus_tag: D1606_09265<br>2) QWUA01000024.1 – locus_tag: D1606_09270<br>3) QWUA01000012.1 - locus_tag: D1606_06200	1) QGST01000005.1 - locus_tag: DK867_07700
chrR	Cr(VI) reductase	1) QWTZ01000005.1 – locus_tag D1604_06960	1) QWUA01000028.1 - locus_tag: D1606_10430<br>2) QWUA01000009.1 - locus_tag: D1606_04965<br>3) QWUA01000055.1 – locus_tag: D1606_13695	1) QGST01000003.1 – locus_tag: DK867_05445<br>2) QGST01000032.1 – locus_tag: DK867_21290<br>3) QGST01000049.1 – locus_tag: DK867_23150
czcD	Export of Cd(II), Co(II), and Zn(II)	1) QWTZ01000005.1 - locus_tag: D1604_07150<br>2) QWTZ01000011.1 - locus_tag: D1604_11025	1) QWUA01000008.1 – locus_tag: D1606_04560	1) QGST01000031.1 - locus_tag: DK867_21070
fieF	Export of Cd(II), Co(II), Fe(II), Ni(II), and Zn(II)	1) QWTZ01000004.1 – locus_tag: D1604_06130	1) QWUA01000006.1 – locus_tag: D1606_03085	1) QGST01000005.1 – locus_tag: DK867_07870
cadA	Export of Cd(II), Co(II) and Zn(II)	-	-	1) QGST01000025.1 – locus_tag: DK867_19895
zntA	Export of Cd(II), Co(II), Cu(II), Ni(II), Pb(II), and Zn(II)	1) QWTZ01000005.1- locus_tag: D1604_07430	-	1) QGST01000032.1 – locus_tag: DK867_21330
zntA	Export of Cd(II), Co(II), Cu(II), Ni(II), Pb(II), and Zn(II)	-	-	1) QGST01000034.1 – locus_tag: DK867_21555
zntA	Export of Cd(II), Co(II), Cu(II), Ni(II), Pb(II), and Zn(II)	-	-	1) QGST01000065.1 – locus_tag: DK867_23595
zntA	Export of Cd(II), Co(II), Cu(II), Ni(II), Pb(II), and Zn(II)			1) QGST01000001.1 – locus_tag: DK867_00450
cadA	Export of Cd(II), Co(II) and Zn(II)	-	-	1) QGST01000004.1 – locus_tag: DK867_06090
yieF	Cr(VI) reductase	1) QWTZ01000002.1 – locus_tag: D1604_04585	1) QWUA01000001.1 – locus_tag: D1606_00205<br>2) QWUA01000055.1 – locus_tag: D1606_13695	1) QGST01000004.1 – locus_tag: DK867_06700
copA	Cu(II) binding and sequestration	1) QWTZ01000002.1 -locus_tag: D1604_04040	1) QWUA01000029.1 – locus_tag: D1606_10605	1) QGST01000001.1 – locus_tag: DK867_01360
cnrA/nccA	Protein participating in forming of CnrABC transmembrane complex exporting Co(II) Ni(II)	1) QWTZ01000002.1 – locus_tag: D1604_04795<br>2) QWTZ01000005.1 – locus_tag: D1604_07170	-	-
cnrB/nccB	Protein participating in forming of CnrABC transmembrane complex exporting Co(II) Ni(II)	1) QWTZ01000005.1 – locus_tag: D1604_07165	-	-
cnrC/nccC	Protein participating in forming of CnrABC transmembrane complex exporting Co(II) Ni(II)	1) QWTZ01000005.1 – locus_tag: D1604_07160<br>2) QWTZ01000002.1 – locus_tag: D1604_04785	-	-
cnrH	RNA polymerase sigma factor activating cnr/ncc genes expression	1) QWTZ01000001.1 – locus_tag: D1604_00685	-	-
nccH	RNA polymerase sigma factor activating cnr/ncc genes expression	1) QWTZ01000004.1 – locus_tag: D1604_05255	-	-
nccN	Component of the NCC cation-efflux system (nccYXHCBAN) that confers resistance to Ni(II), Co(II) and Cd(II). Ion export through a transmembrane tunnel.	1) QWTZ01000005.1 – locus_tag: D1604_07180<br>2) QWTZ01000002.1 – locus_tag: D1604_04195	-	-
pbrA	Component of the prbABC system, conferring resistance to lea	1) QWTZ01000005.1 – locus_tag: D1604_07185	-	-
pbrB/C	Component of the prbABC system, conferring resistance to lea	1) QWTZ01000007.1 – locus_tag: D1604_08845	-	-
